# From interstitial cystitis to chronic pelvic pain

**Published:** 2010-05-25

**Authors:** C Persu, V Cauni, S Gutue, I Blaj, V Jinga, P Geavlete

**Affiliations:** * Urology Department, ‘Saint John’ Clinical Emergency Hospital, BucharestRomania; ** Department of Urology, ‘Th. Burghele’ Clinical Hospital, BucharestRomania; *** Department of Anesthesiology, Central Military Hospital, BucharestRomania

**Keywords:** chronic pelvic pain, interstitial cystitis, urodynamics

## Abstract

There are still many things to be found out about interstitial cystitis/painful bladder syndrome (IC/PBS) because the pathological processes underlying the condition are not yet elucidated, biological markers of the condition are not yet available, and the type and severity of symptoms can vary, so, clearly defining the condition is not yet possible.

For example, it is not clearly understood whether IC/PBS represents a systemic disease, if it is localized in the bladder, or if it was initially localized in the bladder and it later evolved into a systemic disease. This condition is best managed by using a multidisciplinary approach. Management requires a good integration and knowledge of all pelvic organ systems and other systems including musculoskeletal, neurologic, and psychiatric systems.

## Introduction

Patients with IC/PBS suffer from a ‘silent ordeal’; they often appear healthy but experience unrelenting pain that requires frequent trips to the bathroom, both day and night. They may curtail activities due to an extreme urinary frequency. Sleep deprivation caused by pain and nocturia can lead to fatigue and depression. Many are unable to work in this situation. In addition to the personal costs that IC/PBS exacts, the condition is associated with significant health care costs. Experts estimated the total medical expenditures associated with IC/PBS in the United States to be of 65.9 million dollars in 2000 [1].

**Graph 1:**
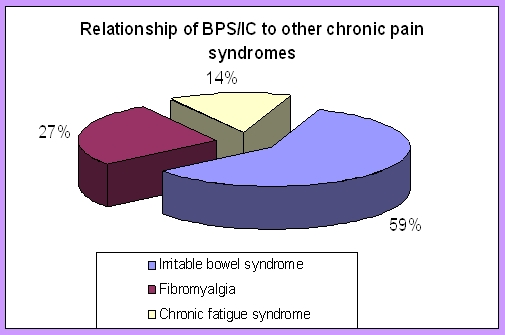
The relationship between BPS/IC and other chronic pain syndromes. [3]

Chronic pelvic pain (known as persistent) occurs for at least 3 months. It is associated with changes in the central nervous system (CNS) that may maintain the perception of pain in the absence of acute injury. These changes may also magnify perception so that non–painful stimuli are perceived as painful (allodynia) and painful stimuli become more painful than expected (hyperalgesia) [2]. 

Curtis Nickel reported data from the Interstitial Cystitis Deep Phenotyping Research Group that confirms the relationship of BPS/IC to other chronic pain syndromes. Irritable bowel syndrome was self–reported in 38.7% of patients vs. 5.6% of controls, fibromyalgia was self–reported in 17.6% of patients vs. 1.7% of controls, and chronic fatigue syndrome in 9.4% vs. 1.1% respectively. No other pain condition was found in 52.4% of the BPS/IC patients studied [3].

PBS/IC includes a major slice of the ‘painful bladder’ disease complex, which consists of a large group of patients with bladder and/or urethral and/or pelvic pain, irritative voiding symptoms (urgency, frequency, nocturia, dysuria), and sterile urine cultures. Painful bladder conditions with well–established causes include radiation cystitis, cystitis caused by microorganisms that are not detected by routine culture methodologies, and systemic disorders that affect the bladder. In addition, many gynecologic disorders can mimic PBS/IC [4].

The perception that the original term, *interstitial cystitis*, was not at all describing the clinical syndrome or even the pathologic findings in many cases has led to the current effort of reconsidering the name of the disorder and even the way it is positioned in the medical spectrum [5]. What was originally considered a bladder disease is now considered a chronic pain syndrome [6] that may begin as a pathologic process in the bladder in most but not all patients and, eventually, it can develop into a disease in a small subset of those affected that even cystectomy may not benefit from [7]. Its relationship to other chronic pelvic pain syndromes including the chronic pelvic pain syndrome in men (previously referred to as nonbacterial prostatitis) is unclear [8].

### Terminology issues

Not only has the definition of IC/PBS been controversial but even the name is under debate. A number of other names have been used to describe this condition, including *interstitial cystitis, painful bladder syndrome, urethral syndrome, trigonitis*, and *bladder pain syndrome*, among others. In recent years, a variety of groups have proposed different names, based in part on their beliefs about the underlying etiology of IC/CBS.

IC was recognized as a pathologic entity during the 19th century. Joseph Parrish, a Philadelphia surgeon, described three cases of severe lower urinary tract symptoms in the absence of a bladder stone in an 1836 paper and termed the disorder ‘tic doloureux of the bladder’ [9]. Teichman and colleagues (2000) argue that this may represent the first description of IC. 

Fifty years later, Skene (1887) used the term *interstitial cystitis* to describe an inflammation that has ‘destroyed the mucous membrane partly or wholly and extended to the muscular parietes’ in his book ‘Diseases of the Bladder and Urethra in Women’. Early in the 20th century, at a New England Section meeting of the American Urological Association, Guy Hunner reported the case of eight women with a history of suprapubic pain, frequency, nocturia, and urgency lasting for an average of 17 years [10]. In Baltimore 1914, he documented on non–trigonal ulcers and bladder epithelial damage associated with interstitial cystitis. He drew attention to the disease, and, the red, bleeding areas he described on the bladder wall came to have the pseudonym ‘Hunner's ulcer.’ 

**Figure 1:**
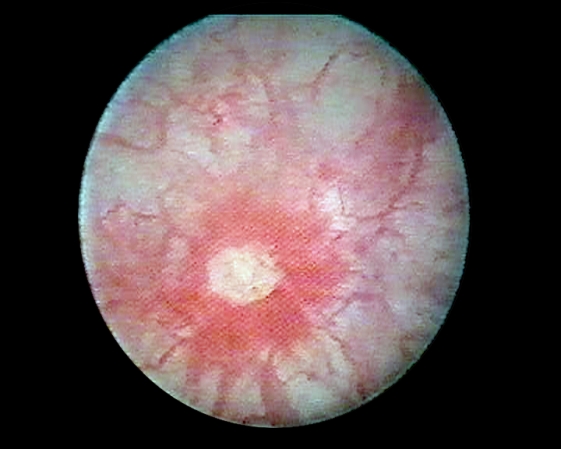
Hunner's ulcer

In 1987, the NIH–NIDDK (National Institutes of Diabetes and Digestive and Kidney Disease) came with the criteria for the diagnosis of interstitial cystitis. This was probably the first structured forma proposal of diagnostic standards in IC.

Although it was proposed some years before, in 2002, The International Continence Society (ICS) defined the term (PBS/IC) as the complaint of suprapubic pain related to bladder filling, accompanied by other symptoms such as increased daytime and night time frequency, in the absence of a proven urinary infection and other obvious pathology. At this time, the ICS stated that the Interstitial Cystitis is a specific diagnosis (part of PBS) and requires confirmation by typical cystoscopic and histological features. Many consultations that took place in the following years discussed all data that became available, but the terminology and algorithms remained unchanged until now.

In February 2007, the Association of Reproductive Health Professionals and the Interstitial Cystitis Association held the Consensus Meeting on Interstitial Cystitis in Washington DC and developed statements concerning the definition and nomenclature of the condition. In their opinion, the nomenclature of IC/PBS may need to change, but change should not be undertaken now because there is insufficient evidence to support it [11]. 

Chronic Pelvic Pain associated with bladder symptoms, described more than 150 years ago, is a common cause of complaint for a large number of women. Probably, the term Bladder Pain Syndrome is the most accurate to describe the CPP associated with the bladder, but the inclusion of IC to the term (BPS/IC) has to be considered. Work is needed to unify terms and promote the use of validated diagnostic tools.

The 2009 Guidelines of the European Association of Urology dedicates 90 pages to the CPP, without changing or improving terminology, but rather concentrating all definitions, diagnostic or treatment algorithms and lots of clinical evidence that support this new approach to a very old disease.

### Epidemiology

Interstitial cystitis/painful bladder syndrome (IC/PBS) predominantly affects women, with an average age at onset of 40 years old. It also affects men and, in rare cases, children [12]. The estimated prevalence of IC/PBS varies considerably, depending on the criteria used in defining the condition and the data collection methods used. Epidemiological studies have used a variety of data collection techniques–application of specific diagnostic criteria, physician diagnosis, patient self–report, and surveying for suggestive symptoms of the condition—with a resulting wide range of estimated prevalence. The National Institute of Diabetes and Digestive and Kidney Diseases (NIDDK) (based on National Health and Nutrition Examination Survey Ⅲ [NHANES Ⅲ]) estimated that 1.2 million women and 82,000 men in the United States have IC/PBS [13]. One study found a prevalence of IC/PBS in the general population of 60 per 100,000, although this is believed to be an underestimate [14]. Indeed, the prevalence in a managed care population was of 197 per 100,000 for women and of 41 per 100,000 for men when diagnosed by using ICD–9 code [15].

In other countries, the prevalence is of 18–450/100.000 women (Finland), 8-16/100.000 (Netherlands) and 4.5/100.000 (Japan) [16]. For the moment, there is no specific data regarding the incidence of this condition in Romania.

**Table 1:** Prevalence estimated for 100.000 persons in different countries [15]

Netherlands	8–16
Finland	18–450
Japan	4.5
Female to Male ratio	9:1

The only definitive risk factor for IC/PBS is female gender: the female–to–male ratio is generally estimated to be of 9:1 [17]. However, in the managed care study mentioned before, this ratio was only of 5:1 [18]. Other risk factors that have been evaluated include heredity and previous urinary tract infection. Recent research suggests that heredity might play a role in the pathogenesis of IC/PBS. A 2004 study found that the prevalence of IC/PBS in females who have a first–degree relative with confirmed IC/PBS is of 1,431 per 100,000. In comparison, the prevalence rate in the general population is estimated to be of about 60 per 100,000 [19]. The 2004 study also found that the prevalence of IC/PBS in first–degree female relatives of patients with IC/PBS confirmed by NIDDK diagnostic criteria was 17 times higher than the rate in the general population [20]. Previous urinary tract infection has been proposed as a possible risk factor for IC/PBS. Current evidence does not support bacterial infection as a risk factor for the condition, although it remains possible that infection serves as a trigger in some patients. [21]

Adults with a prior history of abuse or traumatization demonstrate hypothalamic–pituitary–adrenal (HPA) axis abnormalities, similar to IC/PBS patients. Childhood sexual abuse and physical traumatization are associated with subsequent lifelong risks of chronic pain syndromes [22].

### Pathology

Although the exact cause of PBS/IC is still unknown, several theories exist. No single theory has been proven to explain the symptoms in all cases; thus, many consider PBS/IC to be multifactorial in nature. One widespread hypothesis is that the bladder lining (epithelium) is deficient and therefore permeable to urinary irritants [23]. This theory has been expanded to state that the bladder epithelial glycocalyx is deficient.

In a normal bladder, large, negatively charged molecules called glycoproteins and glycosaminoglycans (GAG) line the epithelium and provide an impermeable barrier. This is considered the main barrier between urine and circulating blood. A defect in the GAG layer allows leakage and absorption of urinary solutes to occur, the major solute being potassium. Ongoing exposure of the bladder wall to potassium causes inflammation, tissue irritation and injury, mast cell degranulation, and sensory nerve depolarization. This process results in symptoms associated with PBS/IC including urinary frequency, urgency, and pain [24]. Recent studies suggest that mast cells, containing histamine–rich granules, are involved in a variety of neuroinflammatory diseases [25]. Mast cell activation and the subsequent histamine release can be stimulated by acetylcholine, stress, and hormonal fluctuations, which may, in turn, play a role in the flares and seasonal symptoms that often occur with PBS/IC (Stanford, 2001).

For example, most patients with vulvodynia have a positive PST and urgency/frequency, indicating pain of bladder origin (IC). IC deserves far greater consideration in the differential diagnosis of patients with vulvodynia [26].

An alternate hypothesis is that an initial insult to the bladder occurs (e.g., a urinary tract infection), which excites sensory nerves located in the bladder wall. This excitation triggers an inflammatory response, or neurogenic inflammation. This in turn releases the neuropeptide substance P, causing the release of mast–cell mediators, histamines, leukotrienes (resulting in local inflammation), cell and tissue damage, and fibrosis. Ultimately, there is a wind–up of the nervous system with neuroplasticity (c–fibers) and visceral allodynia, and pain beyond the bladder occurs [27].

PBS/IC has also been theorized to be an autoimmune condition because some clinical features resemble a disorder of the immune system. Examples include the chronicity, the exacerbations and remissions, and response to immunosuppressants seen in PBS/IC. It also appears that PBS/IC may have a yet unexplained association with autoimmune diseases and pain syndromes such as vulvar vestibulitis, fibromyalgia, Hashimoto's thyroiditis, and irritable bowel syndrome [28].

A recent study [29] showed increased transient receptor potential subtype 1 vanilloid receptor in nerve fibers of the bladder in painful bladder syndrome and a correlation of the pain score with the relative density of transient receptor potential subtype 1 vanilloid receptor nerve fibers. Transient receptor potential subtype 1 vanilloid receptor may have a role in the pathophysiology of painful bladder syndrome and it is a potential target for the novel therapeutic agents.

Another study [30] investigated the distribution of TNF–related apoptosis–inducing ligand (TRAIL) and its receptors in bladder biopsy samples of patients diagnosed with interstitial cystitis and the role of TRAIL in the pathogenesis of interstitial cystitis. Results indicate that TRAIL–R4 is the predominant receptor in the interstitial cystitis inflammation.

The sialic acid content of THP (Tam Horsfall Protein) [42], a critical component of its biological activity, is reduced in patients with IC. N–glycan shows reduced levels of high molecular weight tri– and tetra–antennary sialylated oligosaccharides. These results are supported by quantitative monosaccharide analysis of neutral and amino sugars in patients vs control subjects. THP was isolated from urine samples of 23 patients with IC and 24 control subjects by salt precipitation. The sialic acid contents were measured by using 1,2–diamino–4,5–methylene dioxybenzene–high performance liquid chromatography analysis. For N–glycan profiling, purified THP was treated with peptide: N–glycosidase F to release N–glycans. The purified N–glycans were labeled with 2–aminobenzamide and were profiled by high–pH anion exchange chromatography (HPAEC) with fluorescence detection. The neutral and amino sugars were determined by HPAEC with pulsed amperometric detection. THP in patients with IC has reduced sialylation and overall glycosylation, and by inference, THP has a role in the pathophysiology of IC.

### Diagnosis

Because of the lack of criteria appropriate for use in the clinical setting, the general approach to diagnosis of IC/PBS nowadays tends to be empirical. If other conditions can be excluded, patients with characteristic signs and symptoms are generally treated for presumed IC/PBS. In certain circumstances, some clinicians may choose to evaluate further, with cystoscopy with hydrodistention under general anesthesia, urodynamic studies, or lidocaine instillation. 

The potassium chloride challenge, or Parsons' test, has also been used for diagnosis. This test involves instillation of potassium chloride into the bladder; a positive result is pain and reproduction of IC/PBS symptoms. Because it tests for epithelial permeability, the potassium chloride challenge is not specific for IC/PBS and provides a positive result in other disorders that involve epithelial leakage [31]. The result is positive in virtually all patients with radiation cystitis and bacterial cystitis. The test also misses up to 25 percent of patients with IC/PBS as defined by NIDDK criteria [32]. The potassium chloride challenge is not widely used because of the low sensitivity and specificity, and because it is a painful test to undergo that also requires invasive urinary catheterization. Nevertheless, some experts feel it may be helpful in the subset of patients who have minimal pain.

The diagnosis is generally subject to more rigorous testing in Europe than in North America, where symptoms in the absence of other obvious causes seem to be the gold standard [33].

Cystometry in conscious IC patients generally demonstrates normal function, the exception being a decreased bladder capacity and hypersensitivity. Pain on bladder filling that reproduces the patient's symptoms is very suggestive of IC.

Performing cystoscopy with the patient under anesthesia allows a sufficient distention of the bladder to afford visualization of either glomerulations or Hunner's ulcers. After filling it with H_2_O to 80 cm for 1 to 2 minutes, the bladder is drained and refilled. The terminal portion of the effluent is often blood tinged. Reinspection will reveal the pinpoint petechial hemorrhages that develop throughout the bladder after distention and, they are not usually seen after the examination without anesthesia [34].

Bladder biopsy is necessary only if needed to rule out other etiologies of the patient's symptoms.

Glomerulations are not specific for IC [35], and only when seen in conjunction with the clinical criteria of pain and frequency, can their finding be viewed as significant. While the presence of a Hunner's ulcer has been associated with pain and urinary urgency, neither the findings of bloody irrigating fluid nor glomerulations are strongly associated with any particular symptom in patients in the ICDB [36]. Clinical, urodynamic, and cystoscopic data strongly suggest that the presence of glomerulations does not select out a meaningful difference in patients with symptoms of PBS/IC [37].

As for the markers, the urine antiproliferative factor (APF), identified by Keay, may prove to be the most accurate marker of PBS/IC when confirmed by other centers. It appears to have the highest sensitivity and specificity of the variety of possible markers tested and fits nicely into an etiologic schema [38].

### New horizons in diagnosis

An ultra–performance liquid chromatography–mass spectrometry (UPLC–MS) based on a metabonomic approach was applied to identify a candidate metabolite with an unknown association with the interstitial cystitis (IC). The urinary marker of IC was identified as phenylacetylglutamine (PAGN) by using NMR and MS/MS analysis. In addition, quantitative methods were developed to determine the urinary PAGN levels by using UPLC-UV. The urinary level of PAGN, measured relative to creatinine (Cr), was significantly elevated in IC patients (mean 0.47mg/mg Cr) compared with BC (bacterial cystitis) patients (mean 0.25mg/mg Cr) and HVs (healthy volunteers) (mean 0.11mg/mg Cr). Interestingly, urinary PAGN/Cr ratios in patients with mild IC (grade Ⅰ) and moderate IC (grade Ⅱ) were higher than for patients with severe IC (grade Ⅲ). Moreover, urinary PAGN/Cr ratios with mild and moderate IC patients (mean 0.30mg/mg Cr) were higher than HVs (mean 0.059mg/mg Cr), in the validation set. These findings establish urinary PAGN/Cr ratios as a novel urinary marker of IC, and may contribute to early diagnosis of IC patients [39].

Another recent study [40] aims to investigate the molecular signatures underlying bladder pain syndrome/interstitial cystitis (BPS/IC) by using cDNA microarray. Microarray gene expression profiles are studied in a matched case–control study by using a system of conditional regression modeling. Main findings are summarized as it follows: firstly, a ‘139–gene’ model was discovered to contain high expressions of bladder epithelium, which feature in BPS/IC. Secondly, complex metabolic reactions, including carbohydrate, lipid, cofactors, vitamins, xenobiotics, nucleotide, and amino acid metabolisms, are found to have a strong relationship with bladder smooth muscle contraction through IC status. Thirdly, it was found that the transcriptional regulations of IC–induced bladder smooth muscle contraction status, including the level of contractile force, tissue homeostasis, energy homeostasis, and the development of nervous system. In addition, this study suggested that the mast–cell activation mediated by the high–affinity receptor of Fc episilon RI triggers allergic inflammation through IC status. Such genetic changes, jointly termed ‘bladder remodeling’ can constitute an important long–term consequence of phosphate–buffered saline (PBS)/IC. The success of this innovation has supported the use of microarray–based expression profiling as a single standardized platform for diagnosis of PBS/IC and offering drug discovery.

Examining the urinary bladder mucosa with a flexible cystoscope with the NBI system makes it possible to easily detect ulcers of bladder mucosa and areas with angiogenesis. Therefore, it is considered that the use of a flexible cystoscope with the NBI system is highly practical for the IC/PBS diagnosis [41].

### New directions in PBS/IC treatment

One study evaluated the safety and efficacy of intravesical liposomes, a mucosal protective agent, compared to oral pentosan polysulfate sodium for interstitial cystitis/painful bladder syndrome [43]. A prospective longitudinal study was performed as a result of the effect of 2 independent treatments (intravesical liposomes and oral pentosan polysulfate sodium) in patients with interstitial cystitis/painful bladder syndrome. Ten possible responses (or measures) to treatment were monitored at three time points, including baseline, and weeks 4 and 8. A total of 24 patients with interstitial cystitis/painful bladder syndrome were evaluated in a 1:1 ratio to intravesical liposomes (80 mg/40 cc distilled water) once weekly or to oral pentosan polysulfate sodium (100 mg) 3 times daily for 4 weeks each. No patient had urinary incontinence, retention or infection due to liposome instillation. There were no unanticipated adverse events and no significant worsening of symptoms during follow–up. Statistically significant decreases in urinary frequency and nocturia were observed in each treatment group. Statistically significant decreases in pain, urgency and the O'Leary–Sant symptom score were observed in the liposome group. Decreased urgency in the liposome group had the most profound effect of the ordinal measures.

Each glycosaminoglycan directed treatment seemed beneficial. Liposome intravesical instillation is safe for interstitial cystitis/painful bladder syndrome with potential improvement after 1 course of therapy for up to 8 weeks. Intravesical liposomes achieved efficacy similar to that of oral pentosan polysulfate sodium. Further large–scale placebo controlled studies are needed. Intravesical liposomes appear to be a promising new treatment for interstitial cystitis/painful bladder syndrome.

Another study compared the clinical effectiveness of type A botulinum toxin (BoNT–A) injections followed by hydrodistention (HD) with HD alone, in patients with interstitial cystitis/painful bladder syndrome (IC/PBS). A prospective, randomized study was performed in a urological referral centre [44]. In all, 67 patients with IC/PBS who had failed conventional treatments were enrolled. Of these, 44 patients received suburothelial injection with 200U (15) or 100U (29) of BoNT-A followed by cystoscopic HD 2 weeks later (BoNT–A groups). The control group (23 patients) received the identical HD procedure with no BoNT–A injection. All patients remained on baseline medications of pentosan polysulphate throughout the study. Bladder pain visual analogue scale (VAS), O'Leary–Sant symptom and problem indexes, functional bladder capacity (FBC) and urodynamic variables were measured at baseline and after treatment. Global response assessment was used to evaluate successful treatment response.

**Figure 2:**
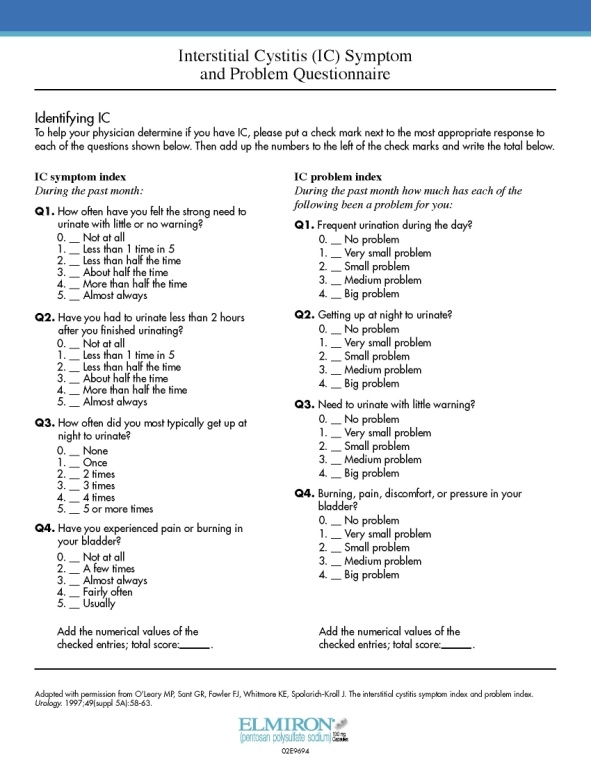
O'Leary–Sant Symptom Score

The IC/PBS symptom score significantly decreased in all three groups, but VAS reduction, FBC and cystometric bladder capacity increases were significant only in the BoNT–A groups at 3 months. Out of the 44 patients in the BoNT–A group 31 (71%) had a successful result at 6 months. A successful result at 12 and 24 months was reported in 24 (55%) and 13 (30%) patients in BoNT–A group, respectively, compared with only six (26%) and four (17%) in the control group (P = 0.002). Intravesical injections of BoNT–A followed by HD produced significantly better clinical results than HD alone in patients with IC/PBS.

Intravesical Alkalinized Lidocaine (PSD597) Offers Sustained Relief from Symptoms of Interstitial Cystitis and Painful Bladder Syndrome. The aim of this study was to assess the immediate and sustained relief of the symptoms of interstitial cystitis/painful bladder syndrome (IC/PBlS) after a consecutive 5–day course of treatment with intravesical alkalinized lidocaine (PSD597), and to characterize the pharmacokinetics of single and multiple doses of intravesical PSD597 in a subgroup of patients. This preliminary study showed that PSD597 was effective in providing sustained amelioration of symptoms of IC/PBlS beyond the acute treatment phase. The drug was safe, well tolerated and devoid of the systemic side effects, often experienced with oral drug administration [45].

Intravesical Nanocrystalline Silver (NCS) Decreases Experimental Bladder Inflammation [46]. Boucher and co–workers from Boston and Dallas examined the effect of intravesical NCS in an established model of bladder inflammation by using protamine sulfate and lipopolysaccharide in the rat. Results showed that short intravesical treatment with 1% NCS significantly decreased protamine/lipopolysaccharide induced urine histamine, bladder explant tumor necrosis alpha–factor, and bladder inflammation including mast cell accumulation and degranulation. NCS inhibited histamine release immediately after pretreatment, but it inhibited the delayed tumor necrosis alpha–factor release even if given after the induction of the inflammatory response. 	

Timely hyaluronan instillation therapy [47] may lead to complete symptom remission or even cure in part of the IC/PBS patients, while some responders need continuous intravesical therapy. The present results suggest that selection of patients for hyaluronan therapy by potassium testing improves the outcome of intravesical therapy with a response rate of > 80%.

Short–Term Results of Bilateral S2–S4 Sacral Neuromodulation are suitable in the Treatment of Refractory Interstitial Cystitis, Painful Bladder Syndrome, and Chronic Pelvic Pain [48]. The efficacy of bilateral caudal epidural sacral neuromodulation for the treatment of refractory chronic pelvic pain (CPP), painful bladder syndrome, and interstitial cystitis (IC) was evaluated. Thirty consecutive patients (21 females, 9 males) with severe refractory symptoms underwent bilateral S2–S4 sacral neuromodulation for CPP/IC. The pain score improved by 40% (p = 0.04) and the UDI–6 (Urogenital Distress Inventory) score by 26% (p = 0.05). On average, patients reported a 42% improvement in their symptoms. SF–36 scores did not improve significantly. In refractory patients, bilateral caudal epidural sacral neuromodulation is another possible mode of treatment, which appears to improve both pelvic pain and voiding symptoms.
